# The impact of the Covid-19 pandemic on work accidents with exposure to biological material in Brazil: an interrupted time series analysis

**DOI:** 10.1590/1980-549720240067

**Published:** 2024-12-16

**Authors:** Luiza Maria Parise Morales, Samara Carolina Rodrigues, Klauss Kleydmann Sabino Garcia

**Affiliations:** I Ministry of Health, Health Surveillance and Environmental Secretariat, Programa de Treinamento em Epidemiologia Aplicada aos Serviços do Sistema Único de Saúde (EPISUS) – Brasília (DF), Brazil.; II Universidade de Brasília, School of Health Sciences – Brasília (DF), Brazil.; III Universidade de Brasília, Tropical Medicine Center, Graduate Program in Tropical Medicine – Brasília (DF), Brazil.

**Keywords:** Interrupted time series analysis, Covid-19 pandemic, Epidemiological monitoring, Surveillance of the workers health

## Abstract

**Objective:**

To analyze the impact of the Covid-19 pandemic on the occurrence of work accidents involving biological materials (ATBio) and to assess changes in the epidemiological profile of these cases.

**Methods:**

An ecological time-series study with a cross-sectional component was conducted using ATBio notifications in Brazil from 2015 to 2022 in the Information System for Notifiable Diseases (Sinan). Interrupted time-series analyses were performed using Prais-Winsten regression models, temporal predictions, and multiple logistic regression to identify changes in the profile between the pandemic and pre-pandemic periods.

**Results:**

A total of 499,916 ATBio cases were recorded between 2015 and 2022, with an increasing trend from 2015 to 2019. During the first year of the pandemic, 57,731 (11.5%) accidents were reported, with an estimated reduction of 791.8 ATBio notifications per month during this period. There was a change in the accident profile, with a higher occurrence of ATBio during the first year of the pandemic among workers with 1 to 8 and 9 to 11 years of education, and a lower occurrence among pregnant women. There was also a higher occurrence of exposure to non-categorized biological materials, increased use of masks and face shields, and higher post-accident seroconversion rates.

**Conclusion:**

The occurrence of ATBio was impacted by the Covid-19 pandemic, leading to a reduction in notifications and a change in the event profile on a national scale.

## INTRODUCTION

Work-related accidents involving exposure to biological material (*acidentes de trabalho com exposição a material biológico* – ATBio) are unexpected incidents that occur in the workplace or work environment, resulting in direct or indirect exposure of a worker to biological material potentially contaminated with pathogens^1^. In Brazil, reporting ATBio cases is mandatory and involves completing an investigation form, which records case-specific information in the Notifiable Diseases Information System (*Sistema de Informação de Agravos de Notificação* – Sinan), managed by the Ministry of Health^1^.

These accidents primarily involve contact with blood through percutaneous exposure^2,3^. However, exposure to mucous membranes, intact skin, or various human or animal biological materials, such as secretions, cerebrospinal fluid, urine, sputum, or saliva, may also qualify as ATBio^1^.

These accidents are more common among healthcare professionals but also affect cleaning, security, and administrative workers^2,4^. Between 2010 and 2016, Brazil reported an average of 95 ATBio cases per day among healthcare workers, with an estimated incidence rate ranging from 14.0 to 16.8 accidents per 1,000 healthcare professionals/year^3,5^. The cases predominantly involve women with a complete secondary education, with nursing technicians and assistants being particularly impacted^4,6^.

One risk related to ATBio is the possibility of transmission of infectious diseases, such as viral hepatitis, HIV/AIDS or respiratory diseases^1^. This risk is monitored by testing — for Hepatitis B, C and HIV — the worker who suffered the accident and, when known, the source individual — who is the individual from whom the biological sample from the accident came^1^.

The COVID-19 pandemic, declared by the World Health Organization (WHO) on March 11^th^, 2020, influenced the patterns of various diseases and health conditions^7,8^. This event stands as one of the most significant in world history, primarily due to the impacts of social isolation, the strain on healthcare professionals and services, and the exacerbation of social inequality indicators^9–11^.

The impact of the pandemic on the epidemiological patterns of diseases and injuries can be quantified using Interrupted Time Series (ITS) analysis, a methodology widely employed internationally for this purpose^12^. Time series consist of sequential observations taken at regular or irregular intervals, aiming to identify patterns and trends over time. Disruptions within a time series may occur due to intentional interventions or events that cause significant shifts in the series’ behavior — such as the COVID-19 pandemic^12^.

Given the pandemic's scale, it is assumed that this event may have altered the patterns and epidemiological profile of these accidents. Therefore, this study aimed to analyze the impact of the COVID-19 pandemic on the occurrence of ATBio and to identify changes in the epidemiological profile of these incidents in Brazil.

## METHODS

### Study design, location, and population

This study is designed as an ecological time series analysis with a cross-sectional analytical component. It utilized data from ATBio notifications across Brazil from January 2015 to December 2022. Only notifications after 2014 were included, as universal reporting of this condition was established in that year^13^. The study population comprised workers aged 14 and older, whether formal or informal, who were documented in the ATBio reporting form.

In analyzing the pandemic's impact, the behavior of the time series was assessed during the initial 12 months of the pandemic (March 2020 to February 2021). The period prior to March 2020 is referred to as the "pre-pandemic" phase, while the period following February 2021 served as a reference point to determine whether the time series behavior returned to expected levels.

### Data source

The anonymized database was requested in accordance with the Access to Information Law via the e-SIC platform, under protocol No. 25072.005288/2023-91, on August 28^th^, 2023. ATBio notifications are sourced from Sinan and the e-SUS Health Surveillance Information System (*Sistema de Informação em Saúde e-SUS Vigilância em Saúde* – e-SUS VS), the official epidemiological surveillance system of the state of Espírito Santo.

### Variables of interest

The following variables were analyzed: a. Sociodemographic: date of ATBio notification; gender; pregnancy status; race/color; education (in years of study); labor market status; and occupation (profession); b. Type of exposure at the time of the accident: percutaneous; intact skin; damaged skin; mucosa; type of biological material involved in the accident (cerebrospinal fluid, liquid or plasma; blood; and others); and whether Personal Protective Equipment (PPE) was used (yes or no); c. PPE, whether the following items were used: mask (yes or no); face shield (yes or no); glasses (yes or no); gloves (yes or no); gown (yes or no); and boots (yes or no); d. Exposure to infectious agents: positive source individual (for HIV, Hepatitis B, or Hepatitis C); positive worker at the time of the accident (for HIV, Hepatitis B, or Hepatitis C); and case outcome (discharge; discharge with serological conversion; abandonment; and death due to ATBio).

### Statistical analysis

Absolute and relative frequency measures of cases were calculated. For the impact analysis, ITS methods were employed^12,14^, which utilize temporal trend analysis to assess the effect of interruptions in the series and quantify their impact.

For this analysis, the number of ATBio notifications was used, applying Prais-Winsten (PW) regression^15,16^, which is recommended for ITS analyses due to its capacity to account for temporal trends and seasonality while correcting for serial autocorrelation (the influence of one observation on the subsequent one). Here, the first 12 months of the COVID-19 pandemic were treated as a breakpoint (step)^16,17^. These analyses were conducted using the "prais" package in R software (version 4.3.0)^18^.

Temporal prediction analyses were also conducted to compare the observed temporal behavior during the COVID-19 pandemic with the expected behavior had pre-pandemic trends continued. For this analysis, the mean, trend, and seasonality attributes of ATBio notifications were considered. Exponential smoothing and Holt-Winters prediction methods were applied, using an additive multiplicative model for seasonality^19^. The Holt-Winters seasonal multiplicative model accounts for a strong seasonal effect on the increase in cases, unlike the additive model, which assumes a weaker seasonal influence^19^.

The Holt-Winters prediction model utilized ATBio notifications recorded up to February 2020, projecting cases for the following 34 months. This approach allowed for a comparison between predicted data and actual ATBio records after February 2020. Consequently, a descriptive analysis was conducted comparing the expected data (estimated by the prediction model) with the total number of observed cases during the first 12 months of the pandemic and the subsequent period.

To identify potential changes in the epidemiological profile of the event during the first year of the COVID-19 pandemic, univariate and multiple logistic regression models were applied^20^. Profile changes were assessed for variables that showed statistically significant results in the adjusted analysis (p < 0.05).

The dependent variable (Y) was defined as the period within the first 12 months of the pandemic (March 2020 to February 2021), labeled as the "pandemic" period. Notifications prior to the pandemic onset (before March 2020) were classified as the "pre-pandemic" period. Data after February 2021 were excluded from the logistic regression analysis, as it is understood that ATBio notifications following this date reflect an altered epidemiological profile due to factors such as the initiation of vaccination and the relaxation of isolation measures.

Variables with a p-value below 0.20 in the univariate model were included in the multiple model. The variable "used PPE" was excluded due to collinearity with other PPE use variables (mask, face shield, gloves, goggles, gown, and boots). The stepwise method was applied to determine the best-fitting model. Selection of the most appropriate multiple model was based on Nagelkerke's pseudo R² metrics and the Akaike Information Criterion. Additionally, variables with a p-value below 0.05 were considered statistically significant. Logistic regression was conducted using Jamovi software (version 2.4.11)^21^.

### Ethical considerations

Since this study uses secondary data that is publicly available and anonymized, ethical review was not required, in accordance with CNS Resolution No. 466/2012.

## RESULTS

**Figure 1:**
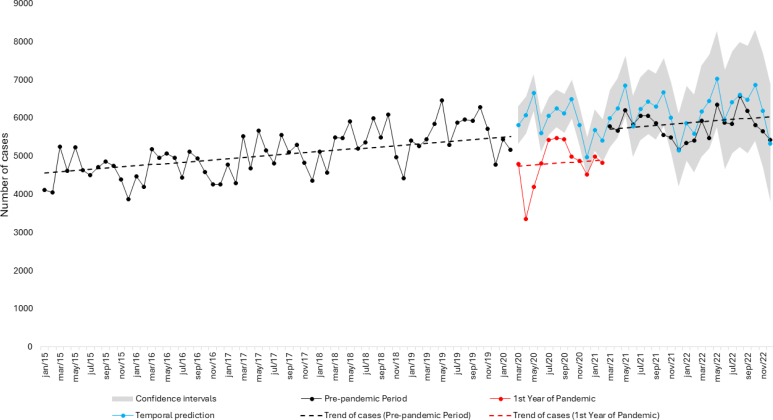
Notifications, trends, and temporal prediction of work accidents involving exposure to biological material in Brazil, 2015 to 2022.

Between January 2015 and February 2022, a total of 499,916 ATBio notifications were identified. Notifications steadily increased until 2019, followed by a decline from March 2020 to February 2021. From 2015 to February 2020, 314,570 ATBio cases were reported, with 68,236 cases recorded in the 12 months prior to the pandemic (March 2019 to February 2020). During the first 12 months of the pandemic (March 2020 to February 2021), 57,731 cases were reported, marking a 15.4% reduction compared to the preceding 12 months. In the subsequent 12 months (March 2021 to February 2022), 68,460 cases were recorded (Figure 1).

The STI analysis estimated a monthly decrease of 791.8 cases (95% CI: −1,154.6 to −428.9; p<0.01) during the first 12 months of the pandemic. Although 57,731 cases were recorded during this period, temporal prediction models projected approximately 71,005 ATBio cases (95% CI: 65,350 to 79,856) for the same timeframe. Beginning in March 2021, the predictive model estimates aligned closely with the actual ATBio notifications, suggesting a return to expected levels (Figure 1).

Among all ATBio notifications from January 2015 to February 2021, the majority involved workers who: were female (76.8%), with 1.5% reported as pregnant; of white race/color (52.0%); with 9 to 11 years of education (42.9%) or 12 years or more (34.2%); in formal employment (75.6%); and employed in the health sector (74.8%), as presented in Table 1. Within healthcare professions, nursing technicians and assistants accounted for the highest incidence of accidents (45.8%), followed by nurses (8.9%) and physicians (8.5%).

**Table 1:** Epidemiological profile of notifications of work accidents involving exposure to biological material in Brazil, from January 2015 to February 2021.

		Pre-pandemic Period	%	1^st^ Year of Pandemic	%	Total	%
Gender							
	Female	340,273	77.0	43,662	75.6	383,935	76.8
	Male	101,870	23.0	14,062	24.4	115,932	23.2
	Ign/blank	42	0.0	7	0.0	49	0.0
Pregnant							
	Yes	5,447	1.2	438	0.8	5,885	1.2
	No	275,431	62.3	35,768	62.0	311,199	62.3
	Does not apply	132,109	29.9	18,044	31.3	150,153	30.0
	Ign/blank	29,198	6.6	3,481	6.0	32,679	6.5
Race/color							
	White	231,358	52.3	28,465	49.3	259,823	52.0
	Non-White	179,328	40.6	25,907	44.9	205,235	41.1
	Ign/blank	31,499	7.1	3,359	5.8	34,858	7.0
Years of study							
	0 years	358	0.1	46	0.1	404	0.1
	1 to 8 years	23,285	5.3	2,731	4.7	26,016	5.2
	9 to 11 years	189,222	42.8	25,397	44.0	214,619	42.9
	12 years or more	151,541	34.3	19,269	33.4	170,810	34.2
	Ign/blank	77,779	17.6	10,288	17.8	88,067	17.6
Employment Situation							
	Formal	333,739	75.5	44,110	76.4	377,849	75.6
	Informal	12,989	2.9	1,766	3.1	14,755	3.0
	Ign/blank	95,457	21.6	11,855	20.5	107,312	21.5
Occupation							
	Other occupation	112,672	25.5	13,177	22.8	125,849	25.2
	Health	329,513	74.5	44,554	77.2	374,067	74.8
Percutaneous exposure							
	No	89,731	20.3	15,123	26.2	104,854	21.0
	Yes	322,975	73.0	37,132	64.3	360,107	72.0
	Ign/blank	29,479	6.7	5,476	9.5	34,955	7.0
Intact skin exposure							
	No	252,033	57.0	32,601	56.5	284,634	56.9
	Yes	123,720	28.0	15,020	26.0	138,740	27.8
	Ign/blank	66,432	15.0	10,110	17.5	76,542	15.3
Damaged skin exposure							
	No	343,119	77.6	43,680	75.7	386,799	77.4
	Yes	23,527	5.3	2,746	4.8	26,273	5.3
	Ign/blank	75,539	17.1	11,305	19.6	86,844	17.4
Mucosal exposure							
	No	324,355	73.4	40,198	69.6	364,553	72.9
	Yes	48,164	10.9	7,078	12.3	55,242	11.1
	Ign/blank	69,666	15.8	10,455	18.1	80,121	16.0
Type of biological material involved in the accident							
	Cerebrospinal fluid, plasma	7,016	1.6	784	1.4	7,800	1.6
	Blood	345,373	78.1	39,804	68.9	385,177	77.0
	Others	40,365	9.1	9,891	17.1	50,256	10.1
	Ign/blank	49,431	11.2	7,252	12.6	56,683	11.3
Use of PPE							
	No	49,140	11.1	2,828	4.9	51,968	10.4
	Yes	358,880	81.2	49,554	85.8	408,434	81.7
	Ign/blank	34,165	7.7	5,349	9.3	39,514	7.9
Use of mask							
	No	200,528	45.3	9,437	16.3	209,965	42.0
	Yes	185,500	42.0	41,191	71.3	226,691	45.3
	Ign/blank	56,157	12.7	7,103	12.3	63,260	12.7
Use of face shield							
	No	341,207	77.2	36,496	63.2	377,703	75.6
	Yes	30,933	7.0	11,755	20.4	42,688	8.5
	Ign/blank	70,045	15.8	9,480	16.4	79,525	15.9
Use of goggles							
	No	288,391	65.2	29,396	50.9	317,787	63.6
	Yes	93,180	21.1	20,008	34.7	113,188	22.6
	Ign/blank	60,614	13.7	8,327	14.4	68,941	13.8
Use of gloves							
	No	82,350	18.6	8,321	14.4	90,671	18.1
	Yes	323,068	73.1	43,672	75.6	366,740	73.4
	Ign/blank	36,767	8.3	5,738	9.9	42,505	8.5
Use of gown							
	No	201,603	45.6	21,609	37.4	223,212	44.6
	Yes	183,048	41.4	28,172	48.8	211,220	42.3
	Ign/blank	57,534	13.0	7,950	13.8	65,484	13.1
Use of boots							
	No	296,769	67.1	36,542	63.3	333,311	66.7
	Yes	73,457	16.6	11,057	19.2	84,514	16.9
	Ign/blank	71,959	16.3	10,132	17.6	82,091	16.4
Source individual positive							
	Not performed/not detected/negative	411,917	93.2	54,515	94.4	466,432	93.3
	Yes	30,268	6.8	3,216	5.6	33,484	6.7
Positive worker							
	Not performed/not detected/negative	329,706	74.6	46,588	80.7	376,294	75.3
	Yes	112,479	25.4	11,143	19.3	123,622	24.7
Case evolution							
	Discharge	229,828	52.0	27,883	48.3	257,711	51.6
	Discharge with serological conversion	8,322	1.9	4,269	7.4	12,591	2.5
	Abandonment	45,270	10.2	4,647	8.0	49,917	10.0
	Death due to ATBio	116	0.0	24	0.0	140	0.03
	Ign/blank	158,649	35.9	20,908	36.2	179,557	35.9
**Total**		442,185	88.5	57,731	11.5	499,916	100.00

In terms of exposure types, 72.0% were percutaneous, 27.8% involved intact skin, 11.1% affected mucous membranes, and 5.3% involved damaged skin. Regarding the biological material involved in the accidents, blood represented 77.0% of the notifications, while liquids, cerebrospinal fluid, or plasma accounted for 1.6%, and "other" materials comprised 10.1% (Table 1).

Regarding the use of PPE, 81.7% of workers reported using at least one type of PPE. Specifically, 73.4% used gloves, 45.3% wore masks, 42.3% utilized gowns, 22.6% wore glasses, 16.9% used boots, and 8.5% employed face shields (Table 1).

Regarding exposure to infectious agents, only 6.7% of known source individuals had positive test results, while 24.7% of injured workers tested positive (for hepatitis B, hepatitis C, or HIV). Among the workers, 51.6% were discharged, 10.0% abandoned the follow-up of the epidemiological investigation, and 2.5% experienced serological conversion. Additionally, 35.9% of this variable was marked as "ignored" or left blank (Table 1).

The multiple logistic regression model indicated that during the pandemic period, there was a change in the notification profile. Specifically, notifications increased among workers with 1 to 8 years of education (OR=1.12; 95%CI 1.02–1.23; p=0.01) and those with 9 to 11 years of education (OR=1.28; 95%CI 1.23–1.32; p<0.01). Conversely, there was a decrease in notifications among pregnant women (OR=0.55; 95%CI 0.46–0.65; p<0.01) and healthcare workers (OR=0.81; 95%CI 0.77–0.86; p<0.01), as shown in Table 2.

**Table 2:** Change in the epidemiological profile between notifications during the first year of the pandemic compared to notifications prior to the Covid-19 pandemic.

Characteristics		Univariate				Multiple model			
		COR	95%CI		p-value	AOR	95%CI		p-value
Gender									
	Male – Female#	1.08	1.05	1.10	**<0.01** *	0.99	0.91	1.07	0.799
Pregnant									
	Does not apply – No#	1.06	1.04	1.08	**<0.01** *	0.95	0.89	1.02	0.185
	Yes – No#	0.55	0.49	0.60	**<0.01** *	0.55	0.46	0.65	**<0.01** *
Race/color									
	Not White – White#	1.28	1.25	1.30	**<0.01** *	1.02	0.98	1.06	0.337
Years of study									
	0 years – 12 years or more#	0.95	0.70	1.30	0.754	0.99	0.53	1.85	0.98
	1 to 8 years – 12 years or more#	0.87	0.83	0.91	**<0.01** *	1.12	1.02	1.23	**0.02** *
	9 to 11 years – 12 years or more#	1.06	1.04	1.08	**<0.01** *	1.28	1.23	1.32	**<0.01** *
Employment Situation									
	Formal – Informal#	0.91	0.87	0.96	**<0.01** *	1.09	1.00	1.20	0.056
Occupation									
	Health – Other occupation#	1.19	1.16	1.21	**<0.01** *	0.81	0.77	0.86	**<0.01** *
Percutaneous exposure									
	Yes – No#	0.66	0.64	0.67	**<0.01** *	0.78	0.74	0.83	**<0.01** *
Intact skin exposure									
	Yes – No#	0.97	0.95	0.99	**<0.01** *	0.83	0.80	0.87	**<0.01** *
Damaged skin exposure									
	Yes – No#	0.98	0.94	1.02	0.344				
Mucosal exposure									
	Yes – No#	1.18	1.15	1.21	**<0.01** *	0.70	0.66	0.75	**<0.01** *
Type of biological material involved in the accident									
	Others – Cerebrospinal fluid, plasma#	2.52	2.33	2.73	**<0.01** *	2.03	1.75	2.35	**<0.01** *
	Blood – Cerebrospinal fluid, plasma#	1.04	0.96	1.12	0.309	1.12	0.97	1.29	0.112
Use of PPE									
	Yes – No#	3.02	2.91	3.14	**<0.01** *				
Use of mask									
	Yes – No#	9.11	8.90	9.33	**<0.01** *	13.37	12.75	14.01	**<0.01** *
Use of face shield									
	Yes – No#	4.66	4.54	4.78	**<0.01** *	2.25	2.14	2.36	**<0.01** *
Use of goggles									
	Yes – No#	2.40	2.35	2.45	**<0.01** *	0.94	0.90	0.99	**<0.01** *
Use of gloves									
	Yes – No#	1.38	1.34	1.41	**<0.01** *	0.66	0.62	0.69	**<0.01** *
Use of gown									
	Yes – No#	1.49	1.46	1.52	**<0.01** *	0.63	0.60	0.66	**<0.01** *
Use of boots									
	Yes – No#	1.31	1.28	1.35	**<0.01** *	0.81	0.77	0.85	**<0.01** *
Source individual positive									
	Yes – Not performed/not detected/negative#	0.75	0.72	0.78	**<0.01** *	0.86	0.80	0.93	**<0.01** *
Positive worker									
	Yes – Not performed/not detected/negative#	0.61	0.59	0.62	**<0.01** *	0.68	0.65	0.71	**<0.01** *
Case evolution									
	Abandonment – Discharge#	0.81	0.79	0.84	**<0.01** *	1.05	1.00	1.11	0.065
	Discharge with serological conversion – Discharge#	9.73	9.25	10.23	**<0.01** *	5.10	4.65	5.60	**<0.01** *
	Death due to ATBio – Discharge#	5.14	2.98	8.87	**<0.01** *	1.50	0.53	4.26	0.449

* Statistical significance (p<0.05);

# reference level. COR: Crude Odds Ratio; AOR: Adjusted Odds Ratio.

Regarding the type and form of exposure, fewer percutaneous accidents were reported (OR=0.78; 95%CI 0.74–0.83; p<0.01), along with decreased exposure to intact skin (OR=0.83; 95%CI 0.80–0.87; p<0.01) and mucous membranes (OR=0.70; 95%CI 0.66–0.75; p<0.01). Conversely, there was an increase in accidents involving other uncategorized organic materials (OR=2.03; 95%CI 1.75–2.35; p<0.01) compared to liquids, cerebrospinal fluid, or plasma (Table 2).

Among the analyzed PPE, there was a decrease in the use of glasses (OR=0.94; 95%CI 0.90–0.99; p<0.01), gloves (OR=0.66; 95%CI 0.62–0.69; p<0.01), gowns (OR=0.63; 95%CI 0.60–0.66; p<0.01), and boots (OR=0.81; 95%CI 0.77–0.85; p<0.01). Conversely, there was an increase in the use of face masks (OR=13.37; 95%CI 12.75–14.01; p<0.01) and face shields (OR=2.25; 95%CI 2.14–2.36; p<0.01), as detailed in Table 2.

Regarding exposure to infectious agents, there was a decrease in the number of source individuals testing positive for hepatitis B, C, or HIV (OR=0.86; 95%CI 0.80–0.93; p<0.01) and in the number of workers testing positive for hepatitis B, C, or HIV at the time of the accident (OR=0.68; 95%CI 0.65–0.71; p<0.01). However, there was an increase in serological conversions (not necessarily for hepatitis B, C, or HIV) at the end of the case follow-up (OR=5.1; 95%CI 4.65–5.60; p<0.01), as detailed in Table 2.

## DISCUSSION

This study demonstrated a significant reduction in ATBio notifications during the first 12 months of the pandemic, with an estimated decline of −791.8 notifications/month. Notable changes in the profile of cases were observed when compared to the pre-pandemic period. During the first year of the pandemic, alterations occurred in the educational characteristics of workers, the type of exposure, and the organic material involved in accidents. Additionally, there was an increase in the use of PPE, such as masks and face shields, as well as an increase in cases progressing to "discharge with seroconversion."

The continuous increase in records from 2015 to 2019 suggests enhanced sensitivity in the surveillance of this condition. However, throughout 2020, there was a noticeable decline in these reports. This reduction was also observed in Brazil for other conditions and diseases during the Covid-19 pandemic^7,8^. Following the first year of the pandemic, the numbers began to rise again, reaching levels comparable to the earlier predictions (Figure 1).

A reduction in work accidents has been reported in other countries^22,23^, while an increase in work-related Covid-19 cases, particularly among health professionals^24,25^, has also been observed. However, it is important to note that the definitions of work accidents and the methods of notification differ between countries, so comparisons should be approached with caution^26^.

However, it is possible that ATBio notifications during the first year of the pandemic also decreased due to workers being away from their jobs. This could be attributed to individuals being classified as at-risk for complications from Covid-19, such as pregnant women and the aged, or due to the establishment of remote work as a social isolation measure^27,28^. The significant decrease in the proportion of pregnant women supports the notion that this demographic was absent from in-person work activities during the pandemic^28^.

In the first half of 2020, there was a reduction in jobs due to the pandemic, particularly in the trade and services sectors, accompanied by an increase in informal work^29^. During this time, recommendations and actions to suspend outpatient services and elective surgeries were also implemented^30^. However, the pandemic resulted in overcrowding of hospital inpatient services, the establishment of exclusive facilities for treating patients suspected or confirmed to have Covid-19^9,10^, increased work overload, and the reassignment of workers to different roles^31^. Additionally, mental and physical strain among health professionals has been reported globally, adversely affecting their performance and increasing their risk of accidents^32^.

In the first year of the pandemic, a higher incidence of ATBio notifications was observed among workers in waste collection, cleaning, and maintenance services in public areas, particularly among those with 1 to 8 years of education. This professional category, which is frequently exposed to biological materials, was particularly impacted during the pandemic due to the increased production of domestic and hospital waste^33,34^.

Among individuals with 9 to 11 years of education, nursing technicians and assistants were prominent, as their services were in high demand during the pandemic. Historically, these educational and occupational categories have been more frequently associated with ATBio notifications^4^. However, during the pandemic, a decrease in notifications was observed among health professionals compared to other occupations.

This trend may be attributed to healthcare professionals having better access to PPE and guidance — despite experiencing increased workloads during the pandemic. Additionally, these professionals were in a state of "constant alert" regarding the risk of infection, whereas other occupations may not have had access to adequate PPE or guidance on its proper use^25,34^.

Accidents involving exposure to "other types of biological material" — which are not categorized — have increased, likely reflecting heightened exposure to situations that pose a risk of Covid-19 infection. Given that Covid-19 is transmitted through droplets, particles, aerosols, or contact followed by touching mucous membranes^35^, the rise in accidents involving these uncategorized biological materials supports the hypothesis of an increase in incidents that carry a risk of Covid-19 transmission. This is particularly relevant as the individual notification form (*ficha de notificação individual* – FNI) does not include options for "droplets" or "aerosols"^36^.

An increasing trend in the use of PPE among ATBio cases in Brazilian healthcare professionals had been previously reported^37^. During the first year of the pandemic, there was a general increase in PPE usage. However, when analyzing each type of PPE individually, a lower likelihood of using items such as goggles, gloves, gowns, and boots was observed, while there was a significant rise in the use of masks and face shields. This increase is likely attributed to the mandatory use of masks in public and private spaces, as well as on public transport, with employers required to provide these items to their workers^38^. It is important to note, however, that the mandatory use of PPE does not ensure sufficient availability or proper utilization of masks or other protective equipment^39^.

However, there was an increase in cases with "discharge with serological conversion" during the period. It is assumed, therefore, that the number of positive source individuals and workers could have been higher if there had been the option of recording exposure to Covid-19 infection at the time of the accident.

Given that the "evolution" field in the FNI is not limited to seroconversions related to hepatitis B, C, or HIV infections, further investigation into the extent of COVID-19 infections within these notifications is warranted. These findings highlight the necessity of adapting the ATBio FNI to include fields that indicate whether testing for diseases beyond HIV and hepatitis was conducted, as well as fields related to the potential transmission of other work-related infections.

Regarding the limitations of the study, the database did not facilitate the identification of seroconversion diagnoses due to the absence of this information in the "case evolution" field. Additionally, the qualitative responses for the variables "other type of exposure" and "other organic material" were not analyzed, preventing quantification of such information.

FNI records for individuals under 14 years of age were also removed from the "age" field, which may have excluded valid records containing errors in that field. However, notifications for children under 14 years represented only 0.7% of the total notifications. Therefore, it is concluded that these potential losses would not significantly impact the results obtained.

The interpretation of the results suggests that the reduction in ATBio notifications during the first year of the pandemic resulted from a combination of factors, including employee absenteeism, enhanced preventive measures against COVID-19, and primarily, underreporting of cases. However, it remains unclear whether this represents underreporting or a genuine decrease in cases, as the resumption of notifications coincided with the rollout of COVID-19 vaccinations in the country and the easing of social isolation measures. Additionally, there was a general reinforcement of the importance of adhering to occupational biosafety protocols during the pandemic, which may have also contributed to the observed reduction in the first year^40^.

Therefore, given that ATBio notifications were affected during the pandemic, resulting in a decrease in notifications and alterations in the event profile on a national level, this study underscores the necessity of enhancing awareness within the care network regarding the importance of notifying this condition. It is essential to improve the completion of investigation forms and broaden strategies aimed at preventing these accidents, focusing on work environments and processes.
