# Supplementary material for: The impact of the Covid-19 pandemic on work accidents with exposure to biological material in Brazil: an interrupted time series analysis
Source: Rev Bras Epidemiol. 2024 Dec 16;27:e240067. doi: 10.1590/1980-549720240067 (PMC11654641; doi:10.1590/1980-549720240067)
Supplement: Translation

# Translation: O impacto da pandemia de Covid-19 nos acidentes de trabalho com exposição a material biológico no Brasil: uma análise de séries temporais interrompidas

**DOI:** 10.1590/1980-549720240067.2

**Authors:** Luiza Maria Parise Morales, Samara Carolina Rodrigues, Klauss Kleydmann Sabino Garcia

**Affiliations:** I Ministério da Saúde, Secretaria de Vigilância em Saúde e Ambiente, Programa de Treinamento em Epidemiologia Aplicada aos Serviços do Sistema Único de Saúde (EPISUS) – Brasília (DF), Brasil.; II Universidade de Brasília, Faculdade de Ciências da Saúde – Brasília (DF), Brasil.; III Universidade de Brasília, Núcleo de Medicina Tropical, Programa de Pós-Graduação em Medicina Tropical – Brasília (DF), Brasil.

**Keywords:** Análise de séries temporais interrompida, Pandemia Covid-19, Vigilância epidemiológica, Vigilância em saúde do trabalhador

## Abstract

**Objetivo:**

Analisar o impacto da pandemia de Covid-19 na ocorrência de acidentes de trabalho com exposição a material biológico (ATBio) e analisar mudanças de perfil epidemiológico desses casos.

**Métodos:**

Estudo ecológico de séries temporais com componente transversal utilizando notificações de ATBio no Brasil entre 2015 e 2022 no Sistema de Informação de Agravos de Notificação (Sinan). Conduziram-se análises de séries temporais interrompidas utilizando modelos de regressão de Prais-Winsten, predições temporais e regressão logística múltipla para verificar mudança de perfil entre os períodos pandêmico e pré-pandêmico.

**Resultados:**

Foram registrados 499.916 ATBio entre 2015 e 2022, com tendência crescente durante os anos de 2015 a 2019. Durante o primeiro ano da pandemia foram registrados 57.731 (11,5%) acidentes, e estimou-se redução de 791,8 notificações de ATBio ao mês nesse período. Houve alteração no perfil dos acidentes, com maior ocorrência de ATBio durante o primeiro ano da pandemia entre trabalhadores de 1 a 8 anos e de 9 a 11 anos de estudo, e menor ocorrência entre gestantes. Houve maior ocorrência de exposição a materiais biológicos não categorizados, maior utilização de máscaras e protetores faciais, bem como maior conversão sorológica pós-acidente.

**Conclusão:**

A ocorrência de ATBio foi impactada pela pandemia de Covid-19, gerando redução de notificações e mudança de perfil do evento em escala nacional.

## INTRODUÇÃO

Acidentes de trabalho com exposição a material biológico (ATBio) são eventos inesperados, ocorridos no ambiente ou durante o trabalho, em que há exposição direta ou indireta do trabalhador a algum material biológico potencialmente contaminado por patógenos^1^. No Brasil, a notificação de ATBio é compulsória, mediante registro da ficha de investigação, que contém informações do caso, no Sistema de Informação de Agravos de Notificação (Sinan), sob gestão do Ministério da Saúde^1^.

Esses acidentes envolvem, em sua maioria, contato com material sanguíneo com exposição percutânea^2,3^. Entretanto, exposições em mucosas ou em peles íntegras ou por diferentes materiais biológicos humanos ou animais, como secreções, líquor, urina, escarro ou saliva também podem configurar um ATBio^1^.

A ocorrência desses acidentes é maior entre profissionais de saúde, mas também afeta trabalhadores da limpeza, da segurança e de setores administrativos^2,4^. Entre 2010 e 2016, no Brasil, observou-se uma notificação média de 95 casos de ATBio por dia entre profissionais de saúde no país, estimando-se um coeficiente de incidência entre 14,0 e 16,8 acidentes para cada 1.000 profissionais de saúde/ano^3,5^. O perfil de casos é majoritariamente entre mulheres com ensino médio completo. Dentre os profissionais da saúde, destacam-se os técnicos e auxiliares de enfermagem^4,6^.

Um risco relacionado aos ATBio é a possibilidade de transmissão de doenças infecciosas, como hepatites virais, HIV/Aids ou doenças respiratórias^1^. Esse risco é monitorado pela testagem — para Hepatite B, C e HIV — do trabalhador que sofreu o acidente e, quando conhecido, do indivíduo-fonte — que é o indivíduo do qual a amostra biológica do acidente é proveniente^1^.

A pandemia de Covid-19, declarada pela Organização Mundial de Saúde (OMS) em 11 de março de 2020, afetou o comportamento de diversas doenças e agravos^7,8^. Esse evento se caracterizou como um dos mais marcantes da história mundial, principalmente pelo isolamento social, pela sobrecarga de profissionais e serviços de saúde e pela intensificação de marcadores de desigualdades sociais^9–11^.

É possível quantificar o impacto da pandemia no comportamento epidemiológico de doenças e agravos por meio de análises de Séries Temporais Interrompidas (STI), metodologia amplamente utilizada internacionalmente para tal finalidade^12^. Séries temporais são conjuntos de observações sequenciais no tempo, com intervalos regulares ou irregulares. O objetivo dessas análises é identificar padrões e tendências ao longo do tempo. A quebra de segmento temporal pode ocorrer devido a intervenções deliberadas ou por eventos que causem modificações significantes no comportamento da série – como a pandemia de Covid-19^12^.

Diante da sua magnitude, entende-se que esse evento pode ter modificado o comportamento desses acidentes e seu perfil epidemiológico. Assim, o objetivo deste estudo é analisar o efeito da pandemia de Covid-19 na ocorrência de ATBio e identificar mudanças no perfil epidemiológico deste evento no Brasil.

## MÉTODOS

### Desenho, local e população de estudo

Trata-se de um estudo ecológico de séries temporais com um componente transversal analítico. O estudo utilizou dados de notificações de ATBio em todo o Brasil entre janeiro de 2015 e dezembro de 2022. Consideraram-se apenas as notificações de ATBio posteriores a 2014, pois nesse ano a notificação desse agravo passou a ser de caráter universal no país^13^. A população de estudo foi de trabalhadores com idade igual ou superior a 14 anos, formais ou informais, notificados na ficha de ATBio.

Na análise de impacto da pandemia verificou-se o comportamento da série temporal durante os 12 meses iniciais da pandemia (março de 2020 a fevereiro de 2021). O período que precede março de 2020 foi denominado "pré-pandêmico", enquanto o posterior a fevereiro de 2021 foi utilizado como referência para verificar se o comportamento da série temporal voltou ao esperado.

### Fonte de dados

O banco de dados, anonimizado, foi solicitado por meio da Lei de Acesso à Informação, via plataforma e-SIC, através do protocolo n° 25072.005288/2023-91 em 28 de agosto de 2023. As notificações de ATBio são provenientes do Sinan e do Sistema de Informação em Saúde e-SUS Vigilância em Saúde (e-SUS VS), sistema oficial de vigilância epidemiológica do estado do Espírito Santo.

### Variáveis de interesse

Foram analisadas as seguintes variáveis: a. Sociodemográficas: data de notificação do ATBio; sexo; gestação; raça/cor; escolaridade (em anos de estudo); situação no mercado de trabalho e ocupação (profissão); b. Tipo de exposição no momento do acidente: percutânea; em pele íntegra; em pele lesada; em mucosa; tipo de material biológico exposto no acidente (líquor, líquido ou plasma; sangue e outros) e se houve utilização de Equipamento de Proteção Individual — EPI (sim ou não); c. EPI, se houve uso de: máscara (sim ou não); protetor facial (sim ou não); óculos (sim ou não); luva (sim ou não); avental (sim ou não) e bota (sim ou não); d. Exposição a agentes infecciosos: indivíduo-fonte positivo (para HIV, Hepatite B ou Hepatite C); trabalhador positivo no momento do acidente (para HIV, Hepatite B ou Hepatite C) e evolução do caso (alta; alta com conversão sorológica; abandono e óbito por ATBio).

### Análises estatísticas

Calculou-se medidas de frequência absoluta e relativa dos casos. Para a análise de impacto utilizou-se métodos de STI^12,14^, metodologia que utiliza análises de tendências temporais para verificar o efeito de interrupções na série e quantificá-las.

Para esta análise utilizou-se o número de notificações de ATBio e aplicou-se a regressão de Prais-Winsten (PW)^15,16^, que é indicada para a condução de análises de STI devido à sua capacidade de considerar os efeitos da tendência e sazonalidade temporal enquanto corrige os efeitos da autocorrelação serial dos dados (que é a influência que uma observação exerce na observação seguinte). Neste caso, analisou-se os primeiros 12 meses da pandemia de Covid-19 como ponto de interrupção (degrau)^16,17^. Para tais análises, utilizou-se o pacote "prais" do software R (versão 4.3.0)^18^.

Também se utilizou análises de predição temporal para permitir uma análise em relação ao comportamento temporal observado durante a pandemia de Covid-19 e o que era esperado caso o comportamento tivesse se mantido como estava. Para a análise de predição foram considerados os atributos de média, tendência e sazonalidade das notificações de ATBio. Utilizou-se métodos de suavização exponencial e predição de Holt-Winters com influência aditiva da sazonalidade no modelo^19^. O modelo multiplicativo sazonal de Holt-Winters considera que a sazonalidade da série tem um forte efeito no aumento de casos, diferente do modelo aditivo, que apresenta uma influência mais fraca^19^.

O modelo de predição Holt-Winters considerou as notificações de ATBio registradas até fevereiro de 2020, e os casos foram preditos para os 34 meses seguintes, permitindo a comparação dos dados preditos com os registros reais de ATBio posteriores a fevereiro de 2020. Assim, foi possível analisar, descritivamente, os dados esperados (estimados pelo modelo de predição) com o total de casos reais observados durante os 12 primeiros meses da pandemia e o período subsequente.

A fim de identificar possíveis mudanças no perfil epidemiológico do evento durante o primeiro ano da pandemia de Covid-19, aplicou-se modelos de regressão logística univariada e múltipla^20^. A mudança de perfil pôde ser verificada entre as variáveis que apresentaram resultados estatisticamente significativos na análise ajustada (p<0,05).

Configurou-se como variável dependente (Y) o período dos primeiros 12 meses da pandemia (março de 2020 a fevereiro de 2021), denominado como período "pandêmico". As notificações que antecedem o início da pandemia (antes de março de 2020) foram classificadas como período "pré-pandêmico". Para a análise de regressão logística não foram incluídos dados posteriores a fevereiro de 2021 por se entender que as notificações de ATBio após esta data já apresentam um perfil epidemiológico diferente do presenciado nos primeiros 12 meses devido a algumas variáveis, tais como o início da vacinação, e de medidas de flexibilização do isolamento.

As variáveis que, no modelo univariado, apresentaram valor p menor que 0,20 foram inseridas no modelo múltiplo. Neste não se incluiu a variável "utilizou EPI" por ser colinear com as demais variáveis de utilização de EPI (máscara, protetor facial, luva, óculos, avental e bota). Utilizou-se o método *stepwise* para encontrar o modelo com melhor ajuste. Para a escolha do modelo múltiplo mais adequado, considerou-se as métricas de pseudo R^2^ de Nagelkerke e o Critério de Informação de Akaike. Ainda, considerou-se estatisticamente significativas as variáveis com valor p menor que 0,05. A regressão logística foi realizada no *software* Jamovi (versão 2.4.11)^21^.

### Considerações éticas

Por se tratar de dados secundários, de domínio público e anonimizados, este trabalho dispensa apreciação Ética, de acordo com a Resolução CNS n° 466/2012.

## RESULTADOS

Foram identificadas 499.916 notificações de ATBio entre janeiro de 2015 e fevereiro de 2022. Observou-se aumento contínuo das notificações até 2019, com um declínio a partir de março de 2020 até fevereiro de 2021. De 2015 a fevereiro de 2020 foram notificados 314.570 casos de ATBio e nos 12 meses prévios ao início da pandemia (março de 2019 a fevereiro de 2020) foram registrados 68.236 casos. Durante os primeiros 12 meses da pandemia (março de 2020 a fevereiro de 2021) foram registrados 57.731 casos, o que equivale a uma redução de 15,4% comparado aos 12 meses anteriores. Nos 12 meses seguintes (março de 2021 a fevereiro de 2022) foram notificados 68.460 casos (Figura 1).

**Figura 1:** Notificações, tendência e predição temporal de acidentes de trabalho com exposição a material biológico no Brasil, 2015 a 2022.

Estimou-se, na análise de STI, uma diminuição de 791,8 casos ao mês (IC95% −1.154,6;-428,9; p<0,01) nos 12 primeiros meses da pandemia. Enquanto nesse período foram registrados 57.731 casos, os modelos de predição temporal indicaram que no mesmo período esperava-se aproximadamente 71.005 casos de ATBio (IC 95%: 65.350; 79.856). A partir de março de 2021 as estimativas provenientes dos modelos preditivos se aproximaram do número de notificações de ATBio, indicando que a quantidade de ATBio voltou a um comportamento normal (Figura 1).

Dentre todas as notificações de ATBio de janeiro de 2015 a fevereiro de 2021, destacaram-se notificações de trabalhadores: do sexo feminino (76,8%), sendo 1,5% delas gestantes; de raça/cor branca (52,0%), com 9 a 11 anos de estudo (42,9%) ou 12 anos ou mais (34,2%); em situação de trabalho formal (75,6%); e da área da saúde (74,8%); conforme Tabela 1. Dentre os profissionais da saúde, a maior ocorrência de acidentes foi entre técnicos e auxiliares de enfermagem (45,8%), enfermeiros (8,9%) e médicos (8,5%).

**Tabela 1:** Perfil epidemiológico das notificações de acidentes de trabalho com exposição a material no Brasil, de janeiro de 2015 a fevereiro de 2021.

		Período Pré-pandemia	%	1° ano de Pandemia	%	Total	%
Sexo							
	Feminino	340.273	77,0	43.662	75,6	383.935	76,8
	Masculino	101.870	23,0	14.062	24,4	115.932	23,2
	Ign/em branco	42	0,0	7	0,0	49	0,0
Gestante							
	Sim	5.447	1,2	438	0,8	5.885	1,2
	Não	275.431	62,3	35.768	62,0	311.199	62,3
	Não se aplica	132.109	29,9	18.044	31,3	150.153	30,0
	Ign/em branco	29.198	6,6	3.481	6,0	32.679	6,5
Raça/cor							
	Branca	231.358	52,3	28.465	49,3	259.823	52,0
	Não branco	179.328	40,6	25.907	44,9	205.235	41,1
	Ign/em branco	31.499	7,1	3.359	5,8	34.858	7,0
Anos de estudo							
	0 anos	358	0,1	46	0,1	404	0,1
	1 a 8 anos	23.285	5,3	2.731	4,7	26.016	5,2
	9 a 11 anos	189.222	42,8	25.397	44,0	214.619	42,9
	12 anos ou mais	151.541	34,3	19.269	33,4	170.810	34,2
	Ign/em branco	77.779	17,6	10.288	17,8	88.067	17,6
Situação no mercado de trabalho							
	Formal	333.739	75,5	44.110	76,4	377.849	75,6
	Informal	12.989	2,9	1.766	3,1	14.755	3,0
	Ign/em branco	95.457	21,6	11.855	20,5	107.312	21,5
Ocupação							
	Outra ocupação	112.672	25,5	13.177	22,8	125.849	25,2
	Saúde	329.513	74,5	44.554	77,2	374.067	74,8
Exposição percutânea							
	Não	89.731	20,3	15.123	26,2	104.854	21,0
	Sim	322.975	73,0	37.132	64,3	360.107	72,0
	Ign/em branco	29.479	6,7	5.476	9,5	34.955	7,0
Exposição pele íntegra							
	Não	252.033	57,0	32.601	56,5	284.634	56,9
	Sim	123.720	28,0	15.020	26,0	138.740	27,8
	Ign/em branco	66.432	15,0	10.110	17,5	76.542	15,3
Exposição pele lesada							
	Não	343.119	77,6	43.680	75,7	386.799	77,4
	Sim	23.527	5,3	2.746	4,8	26.273	5,3
	Ign/em branco	75.539	17,1	11.305	19,6	86.844	17,4
Exposição mucosa							
	Não	324.355	73,4	40.198	69,6	364.553	72,9
	Sim	48.164	10,9	7.078	12,3	55.242	11,1
	Ign/em branco	69.666	15,8	10.455	18,1	80.121	16,0
Tipo de material biológico do acidente							
	Líquido líquor plasma	7.016	1,6	784	1,4	7.800	1,6
	Sangue	345.373	78,1	39.804	68,9	385.177	77,0
	Outros	40.365	9,1	9.891	17,1	50.256	10,1
	Ign/em branco	49.431	11,2	7.252	12,6	56.683	11,3
Uso de EPI							
	Não	49.140	11,1	2.828	4,9	51.968	10,4
	Sim	358.880	81,2	49.554	85,8	408.434	81,7
	Ign/em branco	34.165	7,7	5.349	9,3	39.514	7,9
Uso de máscara							
	Não	200.528	45,3	9.437	16,3	209.965	42,0
	Sim	185.500	42,0	41.191	71,3	226.691	45,3
	Ign/em branco	56.157	12,7	7.103	12,3	63.260	12,7
Uso de protetor facial							
	Não	341.207	77,2	36.496	63,2	377.703	75,6
	Sim	30.933	7,0	11.755	20,4	42.688	8,5
	Ign/em branco	70.045	15,8	9.480	16,4	79.525	15,9
Uso de óculos							
	Não	288.391	65,2	29.396	50,9	317.787	63,6
	Sim	93.180	21,1	20.008	34,7	113.188	22,6
	Ign/em branco	60.614	13,7	8.327	14,4	68.941	13,8
Uso de luva							
	Não	82.350	18,6	8.321	14,4	90.671	18,1
	Sim	323.068	73,1	43.672	75,6	366.740	73,4
	Ign/em branco	36.767	8,3	5.738	9,9	42.505	8,5
Uso de avental							
	Não	201.603	45,6	21.609	37,4	223.212	44,6
	Sim	183.048	41,4	28.172	48,8	211.220	42,3
	Ign/em branco	57.534	13,0	7.950	13,8	65.484	13,1
Uso de bota							
	Não	296.769	67,1	36.542	63,3	333.311	66,7
	Sim	73.457	16,6	11.057	19,2	84.514	16,9
	Ign/em branco	71.959	16,3	10.132	17,6	82.091	16,4
Indivíduo-fonte positivo							
	Não realizado/não detectado/negativo	411.917	93,2	54.515	94,4	466.432	93,3
	Sim	30.268	6,8	3.216	5,6	33.484	6,7
Trabalhador positivo							
	Não realizado/não detectado/negativo	329.706	74,6	46.588	80,7	376.294	75,3
	Sim	112.479	25,4	11.143	19,3	123.622	24,7
Evolução do caso							
	Alta	229.828	52,0	27.883	48,3	257.711	51,6
	Alta com conversão sorológica	8.322	1,9	4.269	7,4	12.591	2,5
	Abandono	45.270	10,2	4.647	8,0	49.917	10,0
	Óbito por ATBio	116	0,0	24	0,0	140	0,03
	Ign/em branco	158.649	35,9	20.908	36,2	179.557	35,9
**Total**		442.185	88,5	57.731	11,5	499.916	100,00

Quanto aos tipos de exposição, 72,0% foram percutâneas, 27,8% em pele íntegra, 11,1% em mucosas e 5,3% em peles lesadas. Quanto ao tipo de material biológico do acidente, sangue contabilizou 77,0% das notificações, líquidos, líquor ou plasma totalizaram 1,6% e "outros" 10,1% (Tabela 1).

Em relação à utilização de EPI, 81,7% dos trabalhadores relataram utilizar pelo menos um tipo de EPI, 73,4% relataram utilizar luva, 45,3% máscara, 42,3% avental, 22,6% óculos, 16,9% bota e 8,5% utilizaram protetor facial (Tabela 1).

Quanto a exposições a agentes infecciosos, indivíduos-fonte conhecidos com testes positivos contabilizaram apenas 6,7%, enquanto 24,7% dos trabalhadores acidentados apresentaram algum tipo de teste positivo (para hepatites B ou C ou HIV); 51,6% dos trabalhadores apresentaram alta; 10,0% abandonaram o acompanhamento da investigação epidemiológica; 2,5% apresentaram conversão sorológica, porém 35,9% dessa variável foi marcada como "ignorada" ou deixada em branco (Tabela 1).

O modelo de regressão logística múltipla apontou que, durante o período pandêmico, houve mudança no perfil de notificações com aumento de notificações entre trabalhadores com 1 a 8 anos de estudo (OR=1,12; IC95% 1,02–1,23; p=0,01) e 9 a 11 anos de estudo (OR=1,28; IC95% 1,23–1,32; p<0,01). Também houve diminuição das notificações entre gestantes (OR=0,55; IC95% 0,46–0,65; p< 0,01) e entre trabalhadores da saúde (OR=0,81; IC95% 0,77–0,86; p<0,01), conforme Tabela 2.

**Tabela 2:** Mudança do perfil epidemiológico entre notificações durante o primeiro ano de pandemia em comparação às notificações anteriores à pandemia de Covid-19.

Variáveis		Univariada				Modelo múltiplo			
		ORB	IC95%		p-valor	ORA	IC95%		p-valor
Sexo									
	Masculino – Feminino#	1,08	1,05	1,10	**<0,01** *	0,99	0,91	1,07	0,799
Gestante									
	Não se aplica – Não#	1,06	1,04	1,08	**<0,01** *	0,95	0,89	1,02	0,185
	Sim – Não#	0,55	0,49	0,60	**<0,01** *	0,55	0,46	0,65	**<0,01** *
Raça/cor									
	Não-branco – Branca#	1,28	1,25	1,30	**<0,01** *	1,02	0,98	1,06	0,337
Anos de estudo									
	0 anos – 12 anos ou mais#	0,95	0,70	1,30	0,754	0,99	0,53	1,85	0,98
	1 a 8 anos – 12 anos ou mais#	0,87	0,83	0,91	**<0,01** *	1,12	1,02	1,23	**0,02** *
	9 a 11 anos – 12 anos ou mais#	1,06	1,04	1,08	**<0,01** *	1,28	1,23	1,32	**<0,01** *
Situação no mercado de trabalho									
	Formal – Informal#	0,91	0,87	0,96	**<0,01** *	1,09	1,00	1,20	0,056
Ocupação									
	Saúde – Outra ocupação#	1,19	1,16	1,21	**<0,01** *	0,81	0,77	0,86	**<0,01** *
Exposição percutânea									
	Sim – Não#	0,66	0,64	0,67	**<0,01** *	0,78	0,74	0,83	**<0,01** *
Exposição pele íntegra									
	Sim – Não#	0,97	0,95	0,99	**<0,01** *	0,83	0,80	0,87	**<0,01** *
Exposição pele lesada									
	Sim – Não#	0,98	0,94	1,02	0,344				
Exposição mucosa									
	Sim – Não#	1,18	1,15	1,21	**<0,01** *	0,70	0,66	0,75	**<0,01** *
Tipo de material orgânico do acidente									
	Outros – Líquido, Líquor, plasma#	2,52	2,33	2,73	**<0,01** *	2,03	1,75	2,35	**<0,01** *
	Sangue – Líquido, Líquor, plasma#	1,04	0,96	1,12	0,309	1,12	0,97	1,29	0,112
Uso de EPI									
	Sim – Não#	3,02	2,91	3,14	**<0,01** *				
Uso de máscara									
	Sim – Não#	9,11	8,90	9,33	**<0,01** *	13,37	12,75	14,01	**<0,01** *
Uso de protetor facial									
	Sim – Não#	4,66	4,54	4,78	**<0,01** *	2,25	2,14	2,36	**<0,01** *
Uso de óculos									
	Sim – Não#	2,40	2,35	2,45	**<0,01** *	0,94	0,90	0,99	**<0,01** *
Uso de luva									
	Sim – Não#	1,38	1,34	1,41	**<0,01** *	0,66	0,62	0,69	**<0,01** *
Uso de avental									
	Sim – Não#	1,49	1,46	1,52	**<0,01** *	0,63	0,60	0,66	**<0,01** *
Uso de bota									
	Sim – Não#	1,31	1,28	1,35	**<0,01** *	0,81	0,77	0,85	**<0,01** *
Indivíduo-fonte positivo									
	Sim – Não realizado/Não detectado/Negativo#	0,75	0,72	0,78	**<0,01** *	0,86	0,80	0,93	**<0,01** *
Trabalhador positivo									
	Sim – Não realizado/Não detectado/Negativo#	0,61	0,59	0,62	**<0,01** *	0,68	0,65	0,71	**<0,01** *
Evolução do caso									
	Abandono – Alta#	0,81	0,79	0,84	**<0,01** *	1,05	1,00	1,11	0,065
	Alta com conversão sorológica – Alta#	9,73	9,25	10,23	**<0,01** *	5,10	4,65	5,60	**<0,01** *
	Óbito por ATBio – Alta#	5,14	2,98	8,87	**<0,01** *	1,50	0,53	4,26	0,449

* Significância estatística (p<0,05);

# nível de referência. ORB: *Odds Ratio* bruta; ORA: *Odds Ratio* ajustada.

Quanto ao tipo e à forma de exposição, foram notificados menos acidentes percutâneos (OR=0,78; IC95% 0,74–0,83; p<0,01), com exposição a pele íntegra (OR=0,83; IC95% 0,80–0,87; p<0,01) e a mucosas (OR=0,70; IC95% 0,66–0,75; p<0,01). Porém, houve aumento de acidentes com outros materiais orgânicos não categorizados (OR=2,03; IC95% 1,75–2,35; p<0,01) em relação a líquidos, líquor ou plasma (Tabela 2).

Dentro dos EPI analisados, identificou-se menor utilização de óculos (OR=0,94; IC95% 0,90–0,99; p<0,01), luva (OR=0,66; IC95% 0,62–0,69; p<0,01), avental (OR=0,63; IC95% 0,60–0,66; p<0,01) e bota (OR=0,81; IC95% 0,77–0,85; p<0,01). Por outro lado, houve maior utilização de máscaras faciais (OR=13,37; IC95% 12,75–14,01; p<0,01) e protetores faciais (OR=2,25; IC95% 2,14–2,36; p<0,01), conforme Tabela 2.

Quanto à exposição a agentes infeciosos, houve diminuição de indivíduos-fonte testados positivo para hepatites B ou C ou HIV (OR=0,86; IC95% 0,80; 0,93; p<0,01) e de trabalhadores com testes positivos para hepatites B ou C ou HIV (OR=0,68; IC95% 0,65–0,71; p<0,01) no momento do acidente. Porém, no que tange à evolução do caso, houve aumento das conversões sorológicas (não necessariamente para hepatites B ou C ou HIV) ao final do acompanhamento do caso (OR=5,1; IC95% 4,65–5,60; p<0,01), conforme Tabela 2.

## DISCUSSÃO

Este estudo evidenciou que as notificações de ATBio passaram por uma grande redução durante os primeiros 12 meses da pandemia. Para este período a redução estimada foi de −791,8 notificações/mês. Também se identificou alterações significativas no perfil de casos no período quando comparado ao período pré-pandêmico. Durante o primeiro ano de pandemia, houve alterações nas características de escolaridade dos trabalhadores, no tipo de exposição e no tipo de material orgânico do acidente. Também houve aumento na utilização de EPIs do tipo máscara e protetor facial e de evolução para "alta com soroconversão".

O aumento contínuo dos registros entre 2015 e 2019 denota um crescimento da sensibilidade da vigilância desse agravo. Ao longo de 2020 essas notificações diminuíram. No Brasil, essa redução também foi observada para outros agravos e doenças durante a pandemia de Covid-19^7,8^. Após o primeiro ano pandêmico, estas voltaram a crescer, alcançando valores similares às predições realizadas (Figura 1).

Redução de acidentes de trabalho foi registrada também em outros países^22,23^, ao passo que também foi observado aumento de registros de Covid-19 relacionado ao trabalho, em especial entre profissionais de saúde^24,25^. Todavia, cabe ressaltar que as definições de acidente de trabalho e as formas de notificação variam entre os países, e, portanto, comparações devem ser feitas com cautela^26^.

Ainda assim, é possível que as notificações de ATBio durante o primeiro ano da pandemia tenham diminuído também por afastamentos de trabalhadores de seus postos de trabalho, seja por serem considerados grupo de risco para complicações de Covid-19, como gestantes e pessoas idosas, seja pelo estabelecimento de trabalho remoto como medida de isolamento social^27,28^. A diminuição significativa da proporção de gestantes corrobora com o afastamento das atividades laborais presenciais dessa população durante a pandemia^28^.

Na primeira metade de 2020, houve diminuição de postos de trabalho devido à pandemia, em especial nos setores de comércio e serviços, com aumento da informalização do trabalho^29^. Nesse período também foram recomendadas e adotadas suspensões de serviços ambulatoriais e cirurgias eletivas^30^. Entretanto, a pandemia trouxe também superlotação de serviços de internação hospitalar, abertura de serviços exclusivos para atendimento a pacientes suspeitos ou confirmados para infecção por Covid-19^9,10^, sobrecarga de trabalho e remanejamento de trabalhadores para diferentes postos de trabalho^31^. Também se relatou — mundialmente — sobrecarga mental e física dos profissionais da saúde, prejudicando seu desempenho e expondo-os a maior risco de acidentes^31,32^.

No primeiro ano da pandemia, foi observada uma maior ocorrência de ATBio entre trabalhadores dos serviços de coleta de resíduos, limpeza e conservação de áreas públicas, com escolaridade entre 1 e 8 anos de estudo. Essa categoria profissional, recorrentemente exposta a materiais biológicos, foi particularmente afetada durante a pandemia devido ao aumento da produção de lixo doméstico e hospitalar^33,34^.

Entre aqueles com 9 a 11 anos de estudo, destacaram-se técnicos e auxiliares de enfermagem, que foram largamente requisitados durante a pandemia. Essas categorias de escolaridade e ocupação são historicamente mais frequentes nas notificações de ATBio^4^. No entanto, durante a pandemia, foi observada uma diminuição dessas notificações entre os profissionais de saúde, quando comparado a outras ocupações.

Entende-se que isso pode se dever a profissionais da saúde terem maior acesso a EPIs e orientações — mesmo mediante maior carga laboral durante a pandemia —, além do próprio estado de "alerta constante" para o risco de infecção, enquanto outras ocupações podem não ter tido EPI disponíveis ou orientações adequadas de como utilizá-los^25,34^.

Acidentes com exposição a "outros tipos de material biológico" — que não são categorizados — aumentaram, possivelmente sendo um reflexo do aumento de exposição a situações de risco de infecção por Covid-19. Uma vez que é uma infecção transmitida por gotículas, partículas, aerossóis ou contato seguido de toque em mucosa^35^, o aumento de acidentes com "outros tipos de materiais biológicos" diferentes de sangue, líquidos, líquor e plasma corrobora com a hipótese do aumento de acidentes com risco de transmissão de Covid-19, uma vez que não há as opções "gotículas" ou "aerossóis" na ficha de notificação individual (FNI)^36^.

Tendência crescente do uso de EPIs entre casos de ATBio em profissionais de saúde brasileiros já havia sido reportada anteriormente^37^. Durante o primeiro ano de pandemia observou-se aumento no uso de EPIs, em geral. Ao analisar cada EPI individualmente, observou-se uma menor chance de utilização de EPIs do tipo óculos, luva, avental e botas, ao passo que se observou maior uso de máscaras e de protetores faciais durante o primeiro ano da pandemia, provavelmente devido à obrigatoriedade do uso de máscaras para circulação em espaços públicos, privados, em vias e transportes públicos, sendo de obrigação dos empregadores o fornecimento do equipamento a seus trabalhadores^38^. Destaca-se, entretanto, que a obrigatoriedade não é garantia de disponibilidade suficiente ou de uso adequado de máscaras ou outros EPIs^39^.

A diminuição de indivíduos-fonte e trabalhadores positivos, em comparação ao período pré-pandemia, pode se dever ao fato de que a FNI não permite registro de resultados de testes positivos para Covid-19 ou outras doenças respiratórias. Entretanto, houve um aumento de casos com "alta com conversão sorológica" no período. Supõe-se, então, que o número de indivíduos-fonte e trabalhadores positivos poderia ter sido maior caso houvesse a opção de marcar a exposição à infecção por Covid-19 no momento do acidente.

Uma vez que o campo "evolução" na FNI não é restrito a soroconversões de infecções por vírus da hepatite B, C ou HIV, faz-se necessária a investigação mais aprofundada da magnitude de infecções por Covid-19 nessas notificações. Esses achados também sugerem a necessidade de adequação da FNI de ATBio, para que permita a inclusão de campos que indiquem se houve testagem para outras doenças diferentes de infecção por HIV e hepatites, além de incluir também campos que se refiram a possíveis transmissões de outras infecções relacionadas ao trabalho.

Quanto às limitações do estudo, tem-se que o banco de dados não permitiu a identificação do diagnóstico das soroconversões por indisponibilidade dessa informação no campo "evolução do caso". Também não foram analisadas as respostas qualitativas das variáveis "outro tipo de exposição" e "outro material orgânico", não sendo possível quantificar tais informações.

Também retirou-se registros da FNI com idade inferior a 14 anos no campo "idade", o que pode ter excluído registros válidos, mas que continham erro de preenchimento nesse campo. Apesar disso, as notificações de menores de 14 anos representaram 0,7% do total de notificações. Logo, entende-se que essas possíveis perdas não afetariam os resultados encontrados.

A interpretação dos resultados indica que a redução nas notificações de ATBio, durante o primeiro ano da pandemia, deve-se à combinação de vários fatores, como o afastamento do trabalho e o aumento das medidas preventivas contra a Covid-19, bem como, principalmente, à subnotificação dos casos. Entretanto, não é possível afirmar se tratar de subnotificação ou redução real de casos, uma vez que a retomada das notificações se deu concomitantemente à introdução da vacinação contra Covid-19 no país e o início da flexibilização das medidas de isolamento social. Além disso, durante a pandemia houve um reforço geral na importância de se respeitar protocolos de biossegurança de trabalho, fato que também pode ter contribuído para a redução observada no primeiro ano^40^.

Assim, considerando que as notificações de ATBio foram impactadas durante a pandemia, com redução das notificações e mudanças do perfil do evento em escala nacional, aponta-se neste estudo a necessidade de fortalecer a sensibilização da rede de atenção para a notificação desse agravo, qualificar o preenchimento das fichas de investigação e amplificar estratégias de prevenção desses acidentes, com atenção aos ambientes e processos de trabalho.
